# Peer Review of “Finding Potential Adverse Events in the Unstructured Text of Electronic Health Care Records: Development of the Shakespeare Method”

**DOI:** 10.2196/31550

**Published:** 2021-08-11

**Authors:** Mark Antoniou

**Affiliations:** 1 The MARCS Institute for Brain, Behaviour and Development Western Sydney University Penrith Australia

*This is a peer-review report submitted for the paper “Finding Potential Adverse Events in the Unstructured Text of Electronic Health Care Records: Development of the Shakespeare Method”*

## Round 1 Review

### General Comments

This concise manuscript [1] reports an exploratory study that seeks to detect adverse events from the words within electronic health records. By conducting a computational linguistic analysis, the authors aimed to identify patterns of words that can be used to classify such events. The methodology is novel and has potential use cases that could benefit the automation and scalability of applications in the future.

I have some minor comments for the authors to consider:

1. At the end of the *Introduction* section, it would benefit the reader if the authors could provide some justification for why the Shakespeare method might be useful, rather than simply stating “We hoped.”
2. The methods are well described and the results are straightforward.
3. In the *Discussion* section, there are some missing details that should be added. In particular, it would be useful for researchers seeking to follow up on this work to know what lessons were learned during the course of conducting this research. This could take the form of a short limitations paragraph, and importantly, some recommendations to guide future research. Relatedly, some additional details concerning how this work could inform real-world applications would also be welcome.
